# Supplementary material for: No clear evidence for a domain-general violation of expectation effect in the pupillary responses of 9- to 10-month-olds
Source: PLoS One. 2025 Sep 26;20(9):e0332718. doi: 10.1371/journal.pone.0332718 (PMC12469091; doi:10.1371/journal.pone.0332718)
Supplement: S1 File — (DOCX) [file pone.0332718.s001.docx]

**No clear evidence for a domain-general violation of expectation effect in the pupillary responses of 9- to 10-month-olds**

Running title: Domain-general violation of expectation in infancy

**S1. Specification Curve Analysis on pupil dilation**

To investigate whether analytical decisions of our preregistered preprocessing steps and statistical analysis affected the effect of outcome and domain on pupil dilation, we conducted a specification curve analysis by following the steps outlined in Simonsohn and colleagues (2020) (1). First, we defined reasonable analytical decisions for the given dataset (see Table S1 for an overview). Our decisions to include these specific specifications were – if applicable - based on previous papers on specification curve analysis on pupil dilation in infants and specifications used by other researchers (2,3). For instance, some researchers might use 100ms as a baseline, while others prefer to use 500ms, or some researchers define a minimum number of trials that infants need to complete for inclusion in the main analysis, while others do not specify such criteria. By combining all possible analytical paths, a multiverse of potential analyses was created. Note that, as the combination of the different specifications is multiplicative, many potential analyses are created.

**Table S1**

*Analytical decisions included in the preregistered analysis and the specification curve analysis.*

| Processing/ Analysis step | Preregistered Specification | Specifications in the specification  curve analysis |
| --- | --- | --- |
| Interpolation | Yes | Yes, no |
| Filter | Yes | Yes, no |
| Outlier samples |  | Included, excluded |
| Baseline correction | Yes | 100ms, 500ms |
| Valid trials | At least 50% valid samples per trial | At least 50% valid samples per trial, all trials |
| Outlier removal | +/- 3 standard deviations | +/- 3 standard deviations, no removal |
| Analysed time-window |  | 2s - 3.5s, 3.5s - 5s |
| Minimum number of trials to include infant |  | No minimum trials, at least 2 trials for each condition |
| Conditions included | All conditions | All conditions, each condition separately (action, solidity, number, cohesion) |
| Statistical model | Repeated-measures ANOVA | Repeated-measures ANOVA, paired t-test, linear mixed model, linear mixed model with interaction term |

Next, we repeated the analysis of outcome (and domain) on pupil dilation for all potential analyses, which resulted in 3072 different ways in which we could have analyzed the dataset^[[1]](#footnote-1)^. Of these, we can compute the result of the specification for 3058, meaning that 14 analysis paths were rejected, for instance due to too small sample sizes. We find that most of these analyses lead to a non-significant result (N = 2853, 93.30%). However, there were a few analyses which we could have done that would have led to a significant positive (N = 200, 6.54%, i.e., enhanced pupil dilation for unexpected outcomes) or significant negative (N = 5, 0.16%, i.e., enhanced pupil dilation for expected outcomes) effect of outcome on pupil dilation (see Figure S1).

**Figure S1**

*Results of the specification curve analysis.*


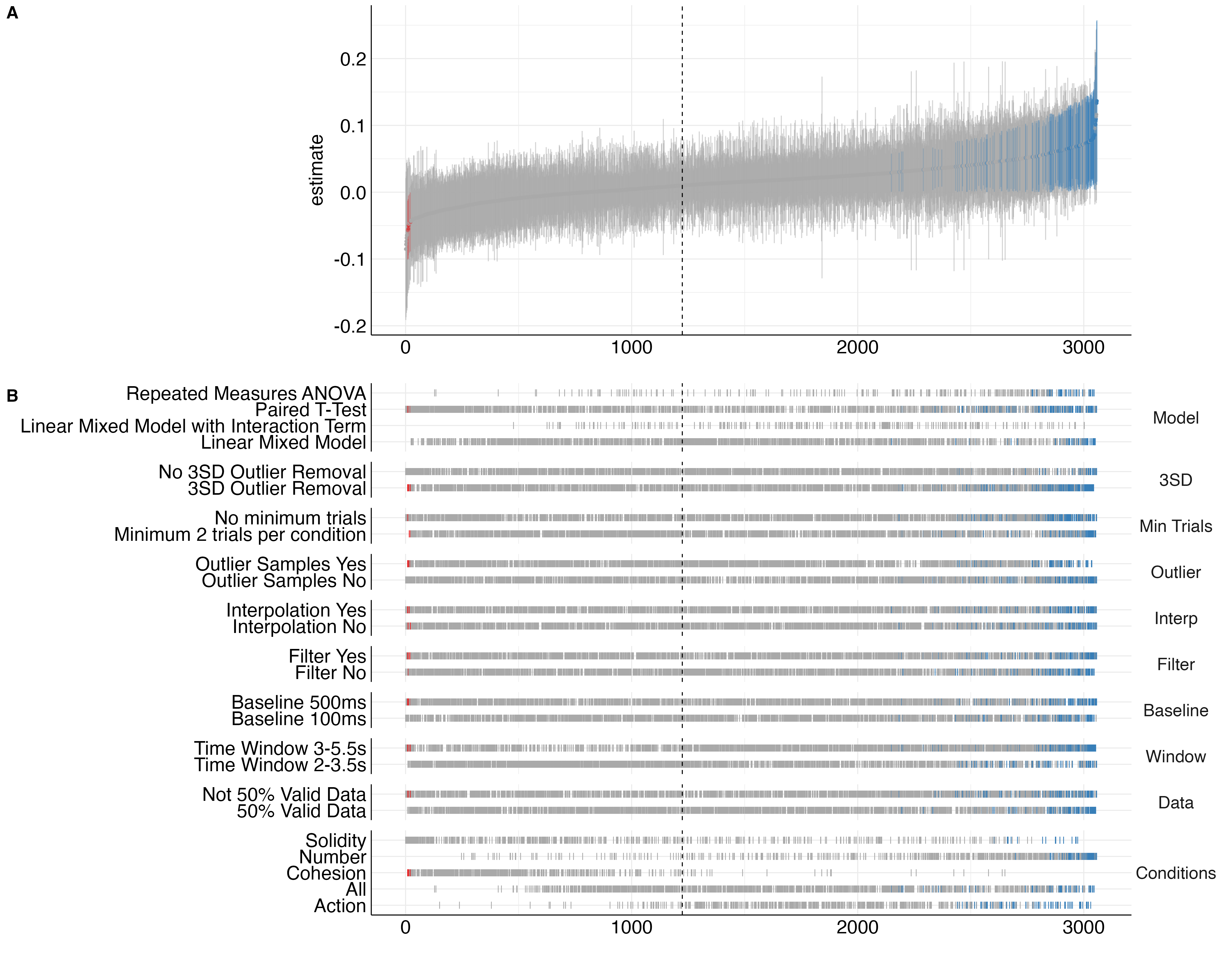


*Note.* In A) the specification curve is displayed with specifications (x-axis) ordered by standardized beta regression estimates and corresponding 95% confidence interval (y-axis). A red color indicates a significant negative result, a blue color a significant positive result, and a grey color a non-significant result. Our preregistered analysis is marked by the dashed black line. In B) the results from the specification curve analysis are displayed separate for each analytical decision.

Last, to investigate whether the results of the specification curve analysis are inconsistent with the null hypothesis of no effect, we conducted a permutation analysis. We created 500 shuffled datasets for which the null hypothesis is true, by randomly shuffling the outcome (expected/unexpected) variable. Thus, there should be no relationship between outcome and pupil dilation. By repeating the specification curve analysis for each of the 500 shuffled datasets, we can get an idea of the results of a specification curve analysis if the null hypothesis is true. By then comparing the shuffled results with our results, we can estimate how inconsistent our results are with the null hypothesis of there being no effect. Following Simonsohn and colleagues (2020) (1), we focused on two different indicators: the 1) median effect size, and 2) the share of significant results. Results of the permutation analysis can be found in Figure S2.

**Figure S2**

*Results of the permutation analysis.*

*
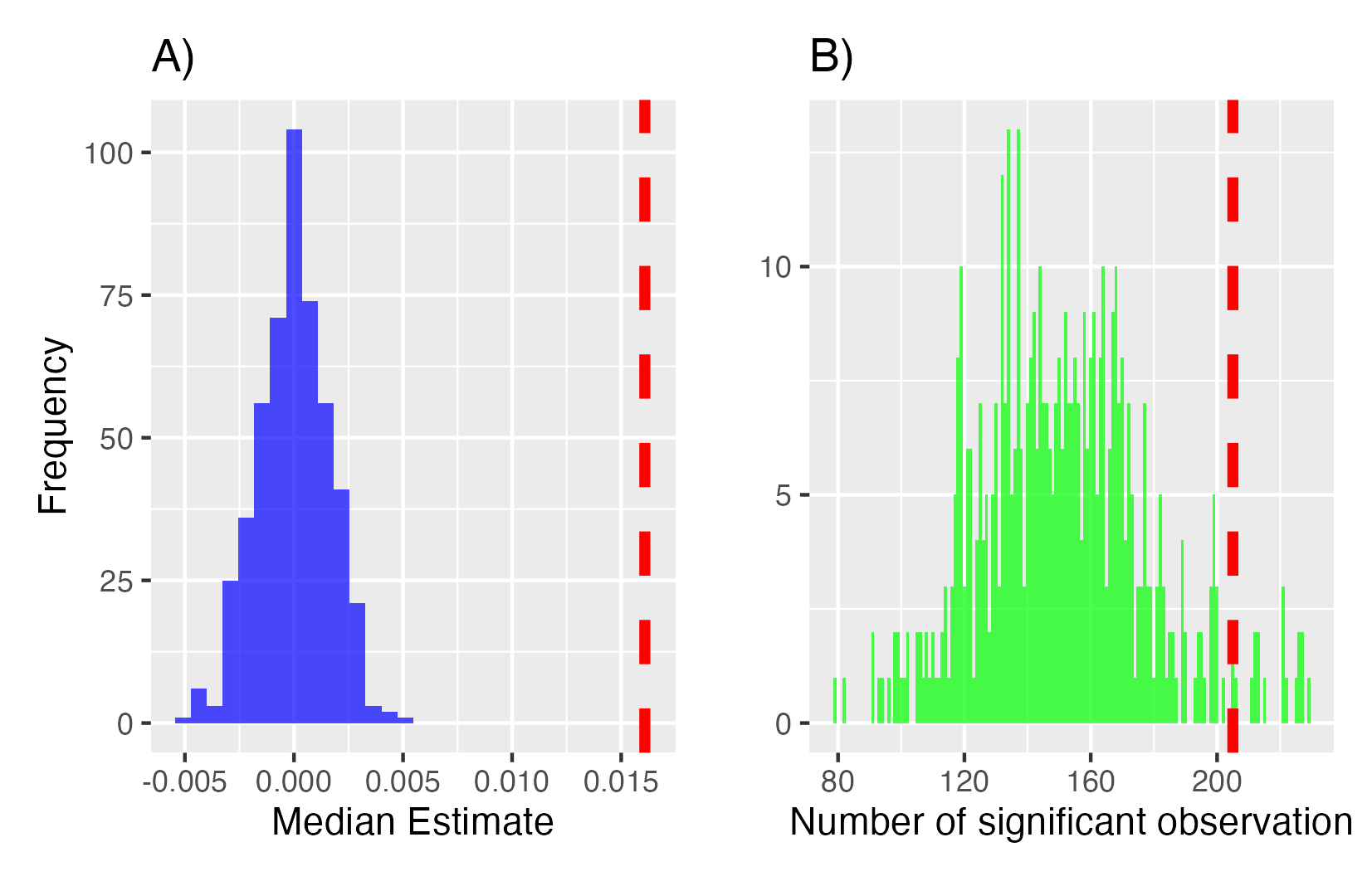
*

*Note.* Results of the permutation analysis compared to the results of the specification curve analysis of the original data, as indicated by the red dashed lined line. A) median estimate. B) Number of significant observations.

We find that results of our dataset slightly differed from the shuffled datasets. No specification computed via the shuffled datasets finds a higher median effect compared to the median effect in our results (Median = .016, *p* < .001). Further, we find that only 17 of the 500 random permutations find more significant results than found in our main analysis (*p* = .034).

To further inspect the permutations, we plotted the specification curve for 6 permutations from the results. We here present the highest, lowest, and median result for each category (median estimate, number of significant observations). The plots can be found in Figure S3.

**
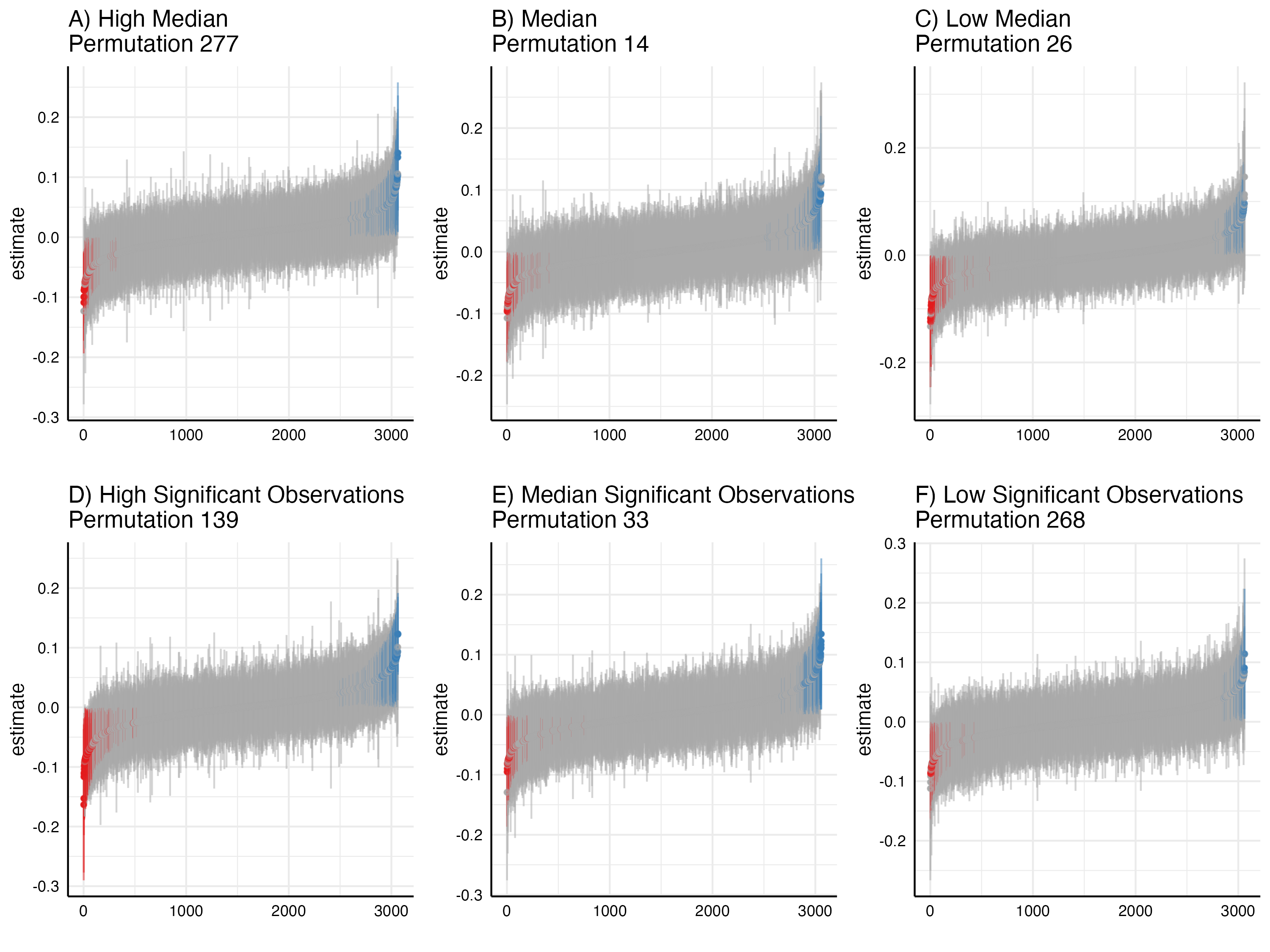
Figure S3**

*Plots of selected specification curve analysis from random permutation data.*

*Note.* Plots of selected specification curve analysis from random permutation data. A-C) shows the random specifications with the highest, median and lowest median estimate. D-F) shows the random specifications with the highest, median and lowest number of significant observations. The similarity of the plots illustrates the similarity in the shuffled data containing little effects and few outliers.

As can be seen in Figure S2, the original data diverged slightly ore from the shuffled data. Similarly, comparing Figure S1 and Figure S3, we can see more positive significant results (blue color) in the original data compared to all specification curve analyses with shuffled data.

Last, we decided to plot the specification curve without confidence intervals for our primary data and for all 500 specifications (Figure S4). Our primary specification (plotted in black) is overall related to a slightly higher estimated value for the effect of outcome on pupil dilation. Still, most results do not reach the level of statistical significance in individual analyses. This might indicate that there is a very small effect of outcome on pupil dilation, compared to a null distribution, but this effect is not large enough to reach statistical significance. Potentially a very large sample may show a statistically significant effect, but it is questionable, whether such small differences provide a meaningful effect.

**Figure S4**

*Comparison of the specification curves for our primary result and the permutations.*


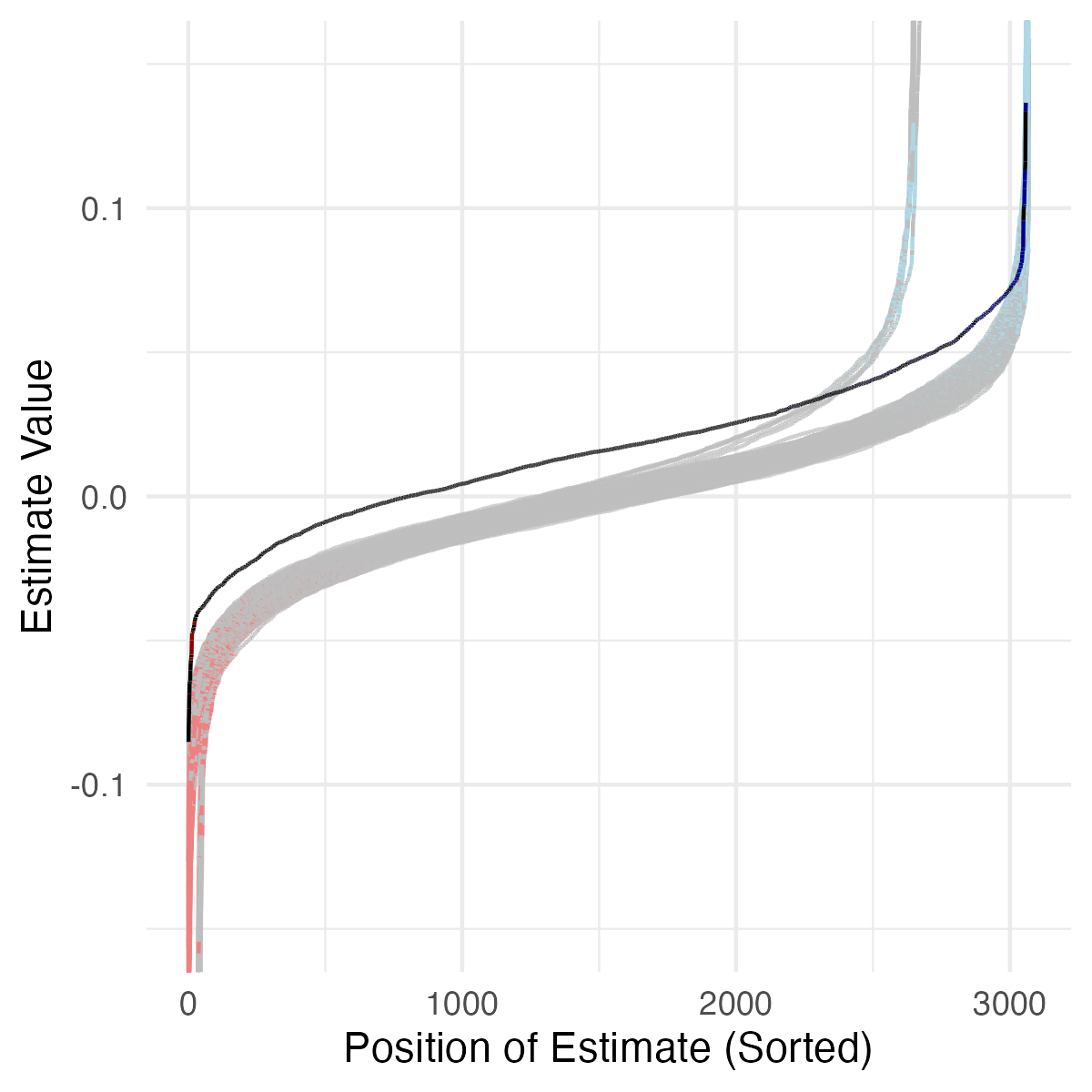


*Note.* Specification curves without confidence intervals for our primary result (black) and the 500 permutations (grey).

**References**

1. Simonsohn U, Simmons JP, Nelson LD. Specification curve analysis. Nature Human Behaviour 2020 4:11 [Internet]. 2020 Jul 27 [cited 2024 Jun 14];4(11):1208–14. Available from: https://www.nature.com/articles/s41562-020-0912-z

2. Calignano G, Girardi P, Altoè G. First steps into the pupillometry multiverse of developmental science. Behav Res Methods [Internet]. 2023 Jul 13 [cited 2024 Apr 28];1:1–20. Available from: https://link.springer.com/article/10.3758/s13428-023-02172-8

3. Sirois S, Brisson J, Blaser E, Calignano G, Donenfeld J, Hepach R, et al. The pupil collaboration: A multi-lab, multi-method analysis of goal attribution in infants. Infant Behav Dev. 2023 Nov 1;73:101890.

1. Note that all combinations as displayed in Table S1 lead to 5120 analysis paths if multiplied. However, not all combinations are possible. For instance, we can only compute a repeated measures ANOVA when also including more than two conditions in the analysis as otherwise the repeated measures ANOVA would be equivalent with the paired t-test. Thus, there are only 3072 possible analysis paths. [↑](#footnote-ref-1)
