# Supplementary material for: No clear evidence for a domain-general violation of expectation effect in the pupillary responses of 9- to 10-month-olds
Source: PLoS One. 2025 Sep 26;20(9):e0332718. doi: 10.1371/journal.pone.0332718 (PMC12469091; doi:10.1371/journal.pone.0332718)
Supplement: S2 File — (DOCX) [file pone.0332718.s002.docx]

**No clear evidence for a domain-general violation of expectation effect in the pupillary responses of 9- to 10-month-olds**

Running title: Domain-general violation of expectation in infancy

**S2. Analysis of looking time data**

In case both eyes were tracked successfully, data of both eyes were averaged. In case only one eye was tracked validly, only data of this eye were used. Only trials in which the infant fixated both priors and the outcome picture at least once were considered for further analyses. Therefore, data were filtered using an IVT-filter with a maximum angle between fixations of 0.5° and a minimum fixation duration of 200 ms (1). Adjacent fixations were merged with a maximum gap of 75 ms. Data were filtered with a moving average of 5 samples and interpolated with a minimum interpolation gap of 10 samples (2). As we applied different trial inclusion criteria for the analysis of looking duration and pupil dilation, a different sample of infants was included in both analyses allowing us to make use of as many data points as possible. Table S2 gives an overview of the sample information for the analysis of looking times.

**Table S2**

*Sample information and information about the amount of included trials per condition in the looking duration analysis.*

| Variable |  |
| --- | --- |
| N | 33 |
| Sex | 20 female |
| Mean age [m.dd] | 9.31 |
| Age range [m.dd – m.dd] | 9.11 – 10.25 |
| Excluded infants: not 2 valid trials | 29 |
| Infants in the sample without additional EEG measurement | 8 |
| Mean included trials action expected/unexpected | 6.36/6.03 |
| Mean included trials cohesion expected/unexpected | 5.63/5.70 |
| Mean included trials number expected/unexpected | 5.58/5.58 |
| Mean included trials solidity expected/unexpected | 6.15/6.24 |

The area of interest covered the whole screen. As preregistered, we performed a 2 x 4 rmANOVA with the within-subject factors outcome (expected vs. unexpected) and domain (action vs. cohesion vs. number vs. solidity) to test whether infants looked longer to the unexpected outcomes as compared to expected outcomes during the 5s presentation of the outcome pictures. This 2 x 4 rmANOVA only revealed a significant main effect of domain, *F*(3,96) = 32.13 , *p* < 0.001, partial η² = 0.50. The main effect of outcome and the interaction were not significant, all *ps* > 0.25 (see Figure S5).

**Figure S5**

*Looking duration per domain and outcome.*


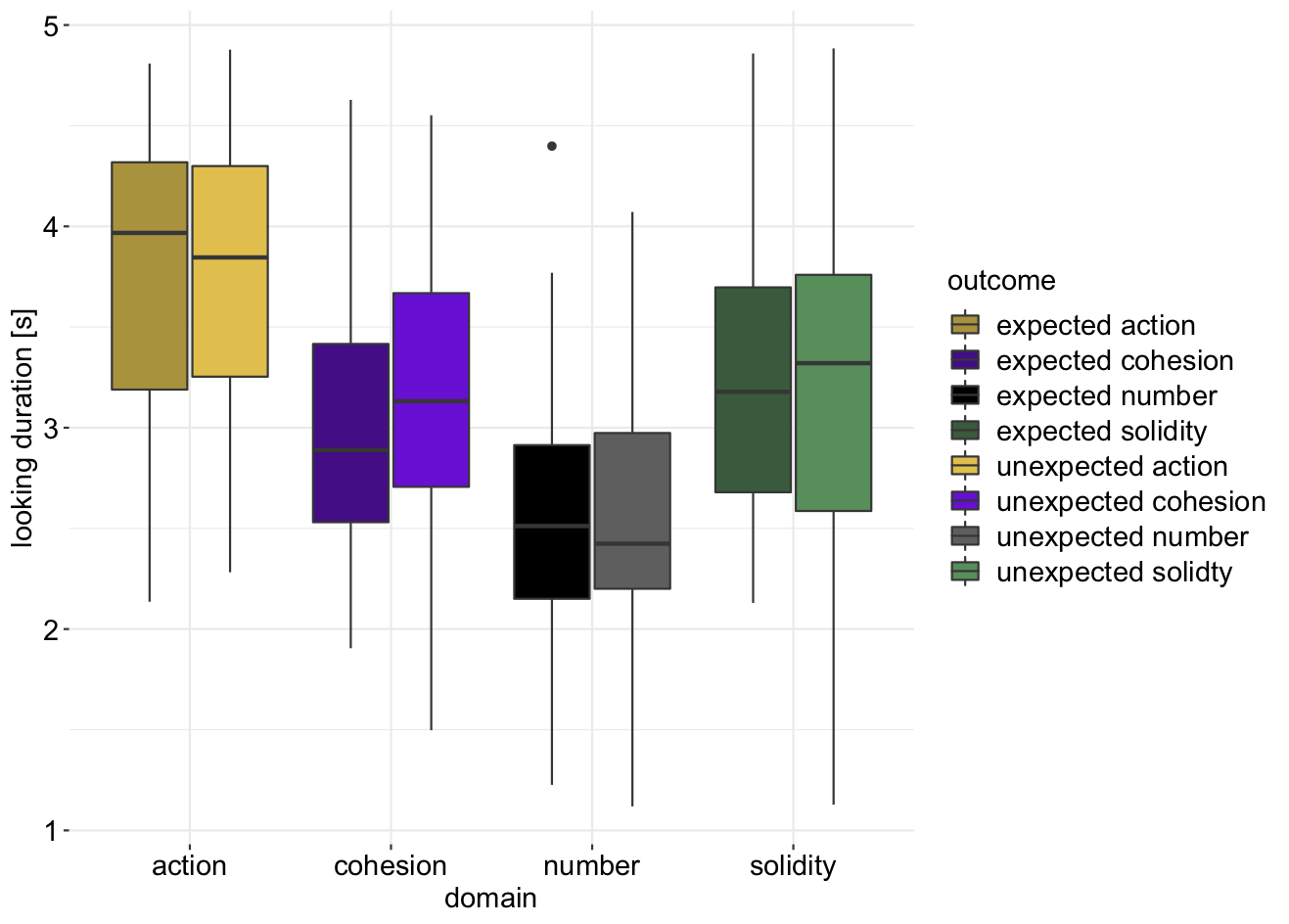


*Note.* Boxplot of looking duration in the four domains for the expected and the unexpected outcome. The horizontal lines reflect the median, the rectangles the range of the first to third quartile and the whiskers the minimum and maximum values. Darker colors (left bar of each domain) represent the expected, lighter colors the unexpected outcomes.

**References**

1. Gredebäck G, Johnson S, von Hofsten C. Eye tracking in infancy research. Dev Neuropsychol [Internet]. 2010;35(1):1–19. Available from: http://www.redi-bw.de/db/ebsco.php/search.ebscohost.com/login.aspx?direct=true&db=psyh&AN=2010-07508-001&site=ehost-live

2. Fawcett C, Wesevich V, Gredebäck G. Pupillary Contagion in Infancy: Evidence for Spontaneous Transfer of Arousal. Psychol Sci. 2016;27(7):997–1003.
